# Supplementary material for: Combining nitric oxide and calcium sensing for the detection of endothelial dysfunction
Source: Commun Chem. 2023 Aug 29;6:179. doi: 10.1038/s42004-023-00973-8 (PMC10465535; doi:10.1038/s42004-023-00973-8)
Supplement: Supplementary file 2 — Supplementary Information [file 42004_2023_973_MOESM2_ESM.pdf]

Supplementary material

Combining nitric oxide and calcium sensing for the detection of endothelial dysfunction

*Valeriia D. Andreeva‡, Haley Ehlers‡, Aswin Krishna R. C.‡, Martin Presselt, Lenie van den Broek, Sylvestre Bonnet**

‡ These authors contributed equally

Content: Equations derivation for Intensometric, ratiometric and pseudo-ratiometric chemosensors.

A. Intensometric sensors

In these calculations, L_0 represents the initial sensor concentration, Ca_0^{2+} the initial calcium ion concentration to be measured, and $[Ca^{2+}]$ the calcium ion concentration at the equilibrium between the calcium ions present locally, and the sensor. We assume a 1:1 binding stoichiometry.

$$Ca^{2+} + L \rightleftarrows CaL$$

$$\boldsymbol{K_d = \frac{[Ca^{2+}][L]}{[CaL]}; [Ca^{2+}] = K_d \frac{[CaL]}{[L]}}$$

Assuming that the emission of the system is linear with the concentration of the free sensor [L] and that of the calcium-bound complex [CaL], we can write

$$\boldsymbol{F = \alpha[L] + \beta[CaL]}$$

In absence of calcium ion

$$F_0 = \alpha L_0$$

And at saturation (in an excess of calcium)

$$F_{max} = \beta[CaL]_{max} = \beta L_0$$

hence,

$$\alpha = \frac{F_0}{L_0}$$

$$\text{and } \beta = \frac{F_{max}}{L_0}$$

$$F = \alpha(L_0 - [CaL]) + \beta[CaL]$$

$$[CaL] = \frac{F - \alpha L_0}{\beta - \alpha}$$

$$\boldsymbol{[CaL] = L_0 \frac{F - F_0}{F_{max} - F_0}}$$

$$F = \alpha[L] + \beta(L_0 - [L])$$

$$[L] = \frac{F - \beta L_0}{\alpha - \beta}$$

$$[L] = L_0 \frac{F - F_{max}}{F_0 - F_{max}}$$

$$\frac{[CaL]}{[L]} = \frac{F - F_0}{F_{max} - F_0} \times \frac{F_0 - F_{max}}{F - F_{max}}$$

$$\frac{[CaL]}{[L]} = \frac{F - F_0}{F_{max} - F}$$

$$[Ca^{2+}] = K_d \frac{F - F_0}{F_{max} - F}$$

$$Ca_0^{2+} = [Ca^{2+}] + [CaL]$$

$$Ca_0^{2+} = K_d \frac{F - F_0}{F_{max} - F} + L_0 \frac{F - F_0}{F_{max} - F_0}$$

Depends on L_0

B. Ratiometric sensors

$$F_1 = \alpha_1[L] + \beta_1[CaL];$$

$$F_{1max} = \cancel{\alpha_1[L]} + \beta_1[CaL] = \beta_1[CaL] = \beta_1 L_0$$

$$F_{10} = \alpha_1[L] + \cancel{\beta_1[CaL]} = \alpha_1[L] = \alpha_1 L_0$$

$$F_2 = \alpha_2[L] + \beta_2[CaL];$$

$$F_{2max} = \alpha_2[L] + \cancel{\beta_2[CaL]} = \alpha_2[L] = \alpha_2 L_0$$

$$F_{20} = \cancel{\alpha_2[L]} + \beta_2[CaL] = \beta_2[CaL] = \beta_2 L_0$$

$$R = \frac{F_1}{F_2};\ R_{max} = \frac{F_{1max}}{F_{20}} = \frac{\beta_1}{\beta_2};\ R_{min} = \frac{F_{10}}{F_{2max}} = \frac{\alpha_1}{\alpha_2}$$

$$R = \frac{F_1}{F_2} = \frac{\alpha_1[L] + \beta_1[CaL]}{\alpha_2[L] + \beta_2[CaL]} = \frac{R_{min}\alpha_2[L] + R_{max}\beta_2[CaL]}{\alpha_2[L] + \beta_2[CaL]}$$

$$\alpha_2[L](R - R_{min}) = \beta_2[CaL](R_{max} - R)$$

$$\frac{[CaL]}{[L]} = \frac{R - R_{min}}{R_{max} - R} \frac{F_{2max}}{F_{20}}$$

$$[CaL]F_{20}(R_{max} - R) = (L_0 - [CaL])F_{2max}(R - R_{min})$$

$$[CaL](F_{20}(R_{max} - R) + F_{2max}(R - R_{min})) = L_0 F_{2max}(R - R_{min})$$

$$[CaL] = L_0 \frac{F_{2max}(R - R_{min})}{F_{20}(R_{max} - R) + F_{2max}(R - R_{min})}$$

$$K_d = \frac{[Ca^{2+}][L]}{[CaL]};\ [Ca^{2+}] = K_d \frac{[CaL]}{[L]}$$

$$\boldsymbol{[Ca^{2+}] = K_d \frac{R - R_{min}}{R_{max} - R} \frac{F_{2max}}{F_{20}}}$$

$$Ca_0^{2+} = [Ca^{2+}] + [CaL]$$

$$\boldsymbol{Ca_0^{2+} = K_d \frac{R - R_{min}}{R_{max} - R} \frac{F_{2max}}{F_{20}} + L_0 \frac{F_{2max}(R - R_{min})}{F_{20}(R_{max} - R) + F_{2max}(R - R_{min})}}$$

Still depends on L_0.

C. Pseudo-ratiometric sensors

$$F_2 = \alpha_2 L_0; L_0 = \frac{F_2}{\alpha_2}$$

$$F_{10} = \alpha_1 L_0 = \frac{\alpha_1}{\alpha_2} F_2$$

$$F_{1max} = \beta_1 L_0 = \frac{\beta_1}{\alpha_2} F_2$$

As for an intensometric sensor:

$$[Ca^{2+}] = K_d \frac{F_1 - F_{10}}{F_{1max} - F_1}$$

$$Ca_0^{2+} = K_d \frac{F_1 - F_{10}}{F_{1max} - F_1} + L_0 \frac{F_1 - F_{10}}{F_{1max} - F_{10}}$$

$$[Ca^{2+}] = K_d \frac{F_1 - \frac{\alpha_1}{\alpha_2} F_2}{\frac{\beta_1}{\alpha_2} F_2 - F_1}$$

$$\boldsymbol{Ca_0^{2+} = K_d \frac{F_1 - \frac{\alpha_1}{\alpha_2} F_2}{\frac{\beta_1}{\alpha_2} F_2 - F_1} + \frac{F_2}{\alpha_2} \times \frac{F_1 - \frac{\alpha_1}{\alpha_2} F_2}{\frac{\beta_1}{\alpha_2} F_2 - \frac{\alpha_1}{\alpha_2} F_2}}$$

Is independent from L_0.

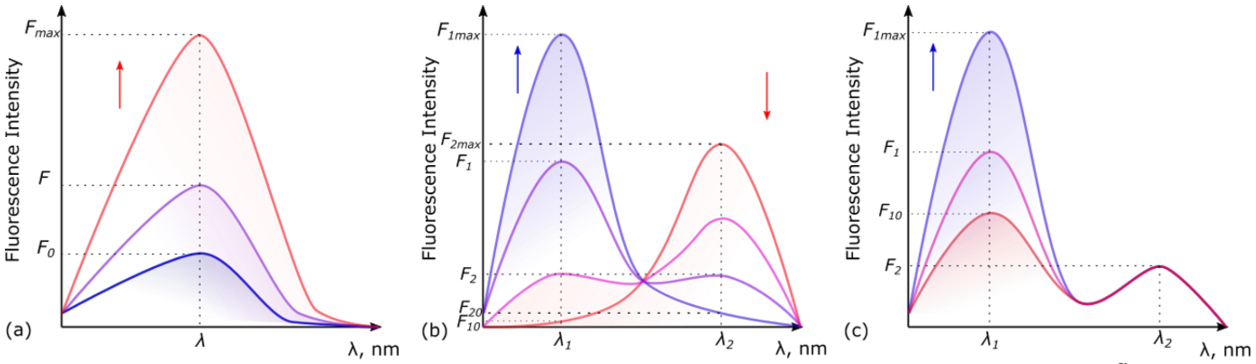

$$[Ca^{2+}] = K_d \frac{F - F_0}{F_{max} - F}$$

$$Ca_0^{2+} = K_d \frac{F - F_0}{F_{max} - F} + L_0 \frac{F - F_0}{F_{max} - F_0}$$

$$[Ca^{2+}] = K_d \frac{R - R_{min}}{R_{max} - R} \frac{F_{2max}}{F_{20}}$$

$$Ca_0^{2+} = K_d \frac{R - R_{min}}{R_{max} - R} \frac{F_{2max}}{F_{20}} + L_0 \frac{F_{2max}(R - R_{min})}{F_{20}(R_{max} - R) + F_{2max}(R - R_{min})}$$

$$[Ca^{2+}] = K_d \frac{F_1 - \frac{\alpha_1}{\alpha_2} F_2}{\frac{\beta_1}{\alpha_2} F_2 - F_1}$$

$$Ca_0^{2+} = K_d \frac{F_1 - \frac{\alpha_1}{\alpha_2} F_2}{\frac{\beta_1}{\alpha_2} F_2 - F_1} + \frac{F_2}{\alpha_2} \times \frac{F_1 - \frac{\alpha_1}{\alpha_2} F_2}{\frac{\beta_1}{\alpha_2} F_2 - \frac{\alpha_1}{\alpha_2} F_2}$$

Figure S1. Typical emission spectra of: (a) intensometric Ca^{2+} chemosensors. $[Ca^{2+}]$– equilibrium Ca^{2+} comcentration, F – fluorescence intencity signal; F_0- fluorescence intensity of Ca^{2+} -free sensor; F_{max} – maximal fluorescence intensity of [CaL] complex; Ca_0^{2+}-Ca^{2+} concentration in the media. (b) ratiometric Ca^{2+} chemosensors. $R = \frac{F_1}{F_2}$ – ratio between fluorescence intensity of [CaL] complex and of Ca^{2+} - free sensor; $R_{max} = \frac{F_{1max}}{F_{20}}$ – ratio while all the sensor molecules been complexed with Ca^{2+} ions; $R_{min} = \frac{F_{10}}{F_{2max}}$ – ratio between fluorescence intensities with absence of Ca^{2+} ions in media. (c) pseudo-ratiometric Ca^{2+} chemosensors. $F_2 = \alpha_2 L_0$ – Ca^{2+} independent fluorescence intensity of the chemosensor; $\frac{\alpha_1}{\alpha_2} = \frac{F_{10}}{F_2}$ – a proportion coefficient of fluorescence intensity of Ca^{2+} -free sensor to Ca^{2+} independent fluorescence intensity of the chemosensor; $\frac{\beta_1}{\alpha_2} = \frac{F_{1max}}{F_2}$ – a proportion coefficient of maximal fluorescence intensity of [CaL] complex to Ca^{2+} independent fluorescence intensity of the chemosensor.
